# Using a Participatory Research Approach in a School-Based Physical Activity Intervention to Prevent Diabetes in the Hualapai Indian Community, Arizona, 2002–2006

**DOI:** 10.5888/pcd11.130397

**Published:** 2014-09-25

**Authors:** Nicolette I. Teufel-Shone, Michelle Gamber, Helen Watahomigie, T. J. Siyuja, Laurie Crozier, Sandra L. Irwin

**Affiliations:** Author Affiliations: Michelle Gamber, University of Arizona, Tucson, Arizona; Helen Watahomigie, T. J. Siyuja Jr, Laurie Crozier, Sandra L. Irwin, Hualapai Tribe, Peach Springs, Arizona.

## Abstract

**Introduction:**

In the United States, type 2 diabetes has reached epidemic proportions among indigenous people. Community-based participatory research offers American Indian communities and university partners an opportunity to integrate skills in community action and systematic inquiry to develop locally acceptable primary prevention interventions to combat diabetes risk factors. The Hualapai Tribe and the University of Arizona designed, implemented, and assessed a school-based physical activity intervention to reduce diabetes risk factors among youth.

**Methods:**

During a 2-year period, trained community members led in-school physical activity classes 2 times per week among students in grades 3 through 8. Body mass index (BMI), fitness measures, and fasting blood glucose level were measured on 6 occasions. Descriptive statistics and *t* tests were used to assess change in outcome measures.

**Results:**

Of the more than 100 youth who took part in the physical activity classes for 2 years, 71 youth (38 male, 33 female) participated in 3 or more data collection sessions. Over time, the percentage of youth with a high fasting blood glucose level of more than 125 mg/dL decreased concurrently with significant improvements in fitness measures. However, BMI increased in both male and female participants. The high number of youth who missed more than 3 data collection sessions was attributed to poor school attendance and tardiness.

**Conclusion:**

Classes led by lay physical activity leaders can affect diabetes risk factors in youth. Incongruous health and fitness outcomes suggest that one indicator does not adequately define the risk profile; BMI alone may not be sufficient as a measure of diabetes risk in youth.

## Introduction

The incidence of childhood obesity and early onset of type 2 diabetes (DM) in American Indian communities has surpassed national rates reported for all races (1–3). High rates of childhood obesity and other DM risk factors has translated into a decreasing age of disease onset in American Indians and an increasing number of DM cases in American Indian children younger than 18 years (3). Compared with the US population as a whole, American Indians are 2.3 times more likely to have DM (4). This trend has serious clinical and public health consequences. The Indian Health Service (IHS) and tribally administered clinics are burdened with the cost of treating early onset secondary complications associated with DM and project that the productivity of young adults will be reduced (5). In response, American Indian communities have used federal, state, and tribal funds to design, implement, evaluate, and refine DM prevention interventions that target youth (6,7).

In 2001, the Health Department of the Hualapai Tribe, located in northwest Arizona, collaborated with the University of Arizona Mel and Enid Zuckerman College of Public Health to launch a community-based participatory research (CBPR) project designed to reduce DM risk factors in elementary school–aged youth. Before this project, the co-principal investigators of the University of Arizona–Hualapai team collaborated for more than 20 years on federally and state-funded health promotion efforts (8). This school-based project yielded lessons valuable to future community–university research efforts related to outcome evaluation and the collection of quantitative data to document longitudinal change.

CBPR is a collaborative process in which lay or professional community members and academically trained researchers partner to integrate their skills in activism and systematic inquiry to develop testable approaches to health problems (8,12). CBPR has been well received by American Indian communities, who have a documented distrust of research, a distrust grounded in past and recent negative portrayals in the academic literature and cases of researchers misinterpreting or disregarding tribal sovereignty and local control of research activities (13,14). The purpose of this study was to examine whether physical activity classes led by lay leaders could change diabetes risk factors among American Indian youth.

## Methods

### Study setting

In 2000, the Hualapai Tribal census reported 2,256 enrolled tribal members (15). The Tribe’s rural 1-million acre reservation is home to approximately 1,600 residents. More than 99% of the community members are either enrolled Hualapai Tribal members or members of other tribes who married into or work in the community. Education and medical services consist of Head Start, an elementary school (kindergarten through 8th grade), a tribally managed emergency medical response unit and health department, and an IHS outpatient clinic. The Tribal Health Department has 40 to 45 staff members and offers behavioral health, medical transport, health education, and support services. The local IHS facility has 8 to 10 clinic-based medical professionals. Specialty services (eg, ophthalmology, podiatry, physical therapy) needed to prevent or treat secondary complications of diabetes are provided approximately 1 time per month by providers from the IHS Colorado River Service Unit, 150 miles to the southwest in Parker, Arizona. Because of limited access to specialty care, the Hualapai Health Department is committed to prevention and building local capacity to teach, promote, and support choices and behaviors associated with a healthy, productive life.

In 2001, the prevalence of diagnosed DM in the Hualapai community was more than 40% in people older than 21 years (16). DM risk factors, such as obesity and low levels of physical activity, are prevalent; more than 85% of the population aged 21 years or older has a body mass index (BMI) of more than 25 kg/m^2^ (16). More alarming is the decreasing age of DM onset; 1 to 2 adolescents in the community are diagnosed with the condition each year (16).

### Intervention

In 2002–2003, the community–university research team completed a 10-month formative assessment of local factors that influence youth wellness (8). This seminal process indicated that sustained, effective youth programs in the community had consistent adult leadership, structured organized activities, and a positive image in and outside the community. Using the Centers for Disease Control and Prevention’s (CDC’s) School Health Index (17), local elementary school personnel identified the absence of an organized physical activity program and low levels of family–school involvement as targets for improvement. Informed by this assessment, the research team developed an in-school physical activity intervention to supplement ongoing communitywide and school break activities. The intervention, known locally as the Youth Wellness Program, was led by 3 project personnel who were community members with knowledge of local physical activity preferences and who were formally trained in the techniques and philosophies of Pathways (18), Physical Best (19), and SPARK (20). One lay physical activity leader has an undergraduate degree in elementary education and the other 2 leaders have some undergraduate course work in physical education or elementary education or both.

The evaluation plan was to track changes in youth’s DM risk factors during their participation in the Youth Wellness Program through the collection of selected fitness and health measures at multiple points during a 2-year intervention. Assessment was limited to youth in grades 3 through 8. Youth in grades kindergarten through 2nd grade were considered too young to provide assent.

With the approval of the local school board and elementary school administration, teachers of grades kindergarten through 8 were offered the opportunity to enroll their entire class in the twice-per-week physical activity program. During the 2-year evaluation phase of the school-based physical activity intervention, youth were allowed to enroll as participants as they newly registered into the school or moved from 2nd grade to 3rd grade. Each activity session lasted 45 to 60 minutes, during which youth engaged in 10 to 15 minutes of stretches, core and upper-body strength training (sit-ups and push-ups), and 35 to 50 minutes of cardiovascular activities. These latter activities were a mix of local favorites (eg, basketball, kickball, softball, flag tag) and SPARK games demonstrated to improve youth cardiovascular strength (20). The protocol was approved by the Hualapai Tribal Council and the University of Arizona institutional review board. All participants provided written minor assent and parental informed consent.

### Study design

The tribe–university research team reviewed a range of potential research outcome measures sufficiently sensitive to assess the effect of the in-school physical activity program. The team’s guiding principles were to select measures that would be appreciated both by tribal and research communities, were locally acceptable, and were feasible for nonmedical personnel to collect in the school environment. The 2-year longitudinal study and chosen assessment measures — BMI, fasting blood glucose level, and a series of physical fitness measures — were presented to and approved by the Tribal Council. Because of the small size of the school and the close-knit nature of the community and school, a randomized control trial was not feasible.

BMI was calculated as weight in kilograms divided by the square of standing height in meters. Standing height was measured with youth in bare or stocking feet and measured in centimeters to the nearest 0.1 cm with a stadiometer (Holtain Ltd). Youth removed jackets, heavy sweatshirts, and shoes, and weight was measured in kilograms to the nearest 0.1 kg using a Toledo self-zeroing weight scale. CDC child BMI-for-age charts (21) were used to categorize participants into 3 weight categories: normal, overweight, and obese. Normal is defined as a BMI from the 5th to less than the 85th percentile, overweight from the 85th to less than the 95th percentile and obese greater than or equal to the 95th percentile (21). All members of the research team were trained by an IHS public health nurse to conduct a finger stick to collect a blood drop sample for the fasting blood glucose level. Blood samples were collected in the morning as youth arrived at school before they ate the school-provided breakfast. Blood glucose was assessed using the FreeStyle Freedom Lite blood glucose meter (Abbott Laboratories Diabetes Care Inc). The American Diabetes Association (ADA) (22) pediatric categories of fasting blood glucose level were used to categorize participants into 3 categories: normal fasting (<100 mg/dL), prediabetes (100–125 mg/dL), and diabetes mellitus (≥126 mg/dL).

The 5 fitness measures, selected from the Cooper Institute’s (23) Fitnessgram, included the PACER (Progressive Aerobic Cardiovascular Endurance Run) run, shuttle run, curl-ups, push-ups, and sit-and-reach. The PACER run, an assessment of cardiovascular fitness, involves continuous running between 2 lines that are 20 meters apart. Using the CD of beep sounds included in Fitnessgram test kit (www.cooperinstitute.org/fitnessgramtests), a youth runs the 20 meters in the time between beeps. Beep spacing decreases with each minute (corresponding to a level), requiring an increase in pace. The youth continues until he or she is unable to keep pace with the beeps for a maximum of 21 levels; the outcome is measured in laps completed (23). The shuttle run, also a measure of cardiovascular fitness, is a 1-mile walk/run measured in the time (in seconds) a youth needs for completion. For curls-up, a measure of body core strength, the youth starts in a supine position with knees bent. The youth curls up and then returns to the supine position; the recorded count is the number of curl-ups completed up to a maximum of 75. For push-ups, a measure of upper arm strength, the youth starts in a prone position with arms straight and hands placed under or slightly wider than the shoulders, legs straight and slightly apart, toes tucked under, and back in a straight line from head to toes throughout the test. The youth lowers his or her body using the arms until the elbows bend at a 90-degree angle and the upper arms are parallel to the floor. This movement is repeated as many times as possible. The count is the number of completed push-ups. The sit-and-reach test, an assessment of flexibility, involves the use of a trunk flexibility box (Baseline Sit-and-reach Trunk Flexibility Box (Fabrication Enterprises, Inc); the top of the box is imprinted with a stretch indicator or scale in inches. The youth removes shoes and sits down, one leg fully extended with the foot flat against the side of the box. The other leg is bent at the knee with the sole of the foot flat on the floor. The arms are extended forward and placed on the top of the box, along the scale with hands placed on top of each other, palms down. The youth reaches forward, sliding his or her hands along the scale 4 times, and then holds the position on the fourth reach for 1 second. The number of inches reached is recorded to the nearest 0.5 inch. 

These measures were easy to implement in the school setting, contributing to their potential sustainability, and could be explained simply to parents and school administrators. Evaluation was conducted on 6 occasions: during the spring and fall of 2004, 2005, and 2006.

### Analysis

Descriptive statistics were used to illustrate group characteristics at points during the intervention, and *t* tests were used to compare physical fitness and biologic measures of the selected participants (those with 3 or more measurement sets) at baseline and at the end of the evaluation phase.

## Results

Of the 10 homeroom classes serving a total school enrollment of 155 to 165 youth, 5 classes participated, yielding an initial sample of 109 students (60 male and 49 female, referred to as *initial participants*) who participated in baseline data collection. After 2 years, 138 children had participated in at least 1 assessment session. Seventy-one of the 138 students participated in 3 or more assessment sessions (referred to as *selected participants*). The students that participated in only 1 or 2 assessment sessions chose to no longer participate, were absent from school that day, were late to school that day, were making up course work during this time, or graduated from the school (“aged-out”).

Paired *t* tests from baseline measurements indicate that characteristics of *initial* and *selected* groups were not significantly different (Table 1). Given the dose–response relationship between physical activity and improved outcome measures (24,25) and the baseline comparability of the 2 groups of participants, only the selected participants were included in further analysis.

**Table 1:** Baseline Characteristics of Initial and Selected Hualapai Youth Participating in a School-Based Intervention to Prevent Diabetes, Arizona, 2004

Characteristic	Initial Participants (N = 109), Mean (Standard Deviation)	Selected Participants (N = 71), Mean (Standard Deviation)	*P* Value^a^
**Age, y**			
Male	10.4 (1.1)	9.9 (0.8)	.63
Female	10.1 (1.4)	9.9 (1.0)	.63
**Body mass index, kg/m^2^**			
Male	24.5 (5.5)	23.8 (4.8)	.50
Female	22.9 (5.5)	21.4 (4.7)	.50
**Fasting blood glucose level, mg/dL**			
Male	109.5 (22.8)	110.5 (43.7)	.78
Female	107.4 (39.3)	110.8 (24.7)	.78

a *P* values determined using *t* test.

No participants in the program were underweight (Tables 2 and 3). Fewer students were classified as normal weight at the end of the evaluation phase and more were classified as overweight and obese, compared with baseline body weight classifications. Compared with baseline fasting blood glucose levels, more children were classified as having normal fasting blood glucose at end of the evaluation phase than at baseline (34.2% vs 46.2% for male participants and 50.6% vs 66.7% for female participants) (Tables 2 and 3). However, more children were classified as having prediabetes at the end of the evaluation (44.7% vs 51.9% for male participants and 24.8% vs 31.1% for female participants). Significantly fewer children were classified as having DM at the end of the evaluation compared with baseline (21.1% vs 1.9% for male participants and 25.6% vs 2.2% for female participants). The results for all fasting plasma glucose categories were significant (*P* = .01).

**Table 2:** Percentage Distribution of Male Body Mass Index (BMI) and Fasting Plasma Glucose Levels (N = 38), Hualapai Youth Participating in a School-Based Intervention to Prevent Diabetes, Arizona, 2004–2006

Measure	Baseline, %	End of Evaluation, %	*P* Value^a^
**Weight classification by BMI percentile**			
Normal (5th to <85th percentile)	29.0	21.2	.67
Overweight (85th to <95th percentile)	18.4	23.0	
Obese (≥95th percentile)	52.6	55.8	
**Classification by fasting glucose level, mg/dL**			
Normal (<100)	34.2	46.2	.01
Prediabetes (100–125)	44.7	51.9	
Diabetes mellitus (≥126)	21.1	1.9	

a *P* values determined using *t* test.

**Table 3:** Percentage Distribution of Female Body Mass Index (BMI) and Fasting Plasma Glucose Levels (N = 33), Hualapai Youth Participating in a School-Based Intervention to Prevent Diabetes, Arizona, 2004–2006

Measure	Baseline, %	End of Evaluation, %	*P* Value^a^
**Weight classification by BMI percentile**			
Normal (5th to <85th percentile)	42.3	28.9	.46
Overweight (85th to <95th percentile)	24.3	28.9	
Obese (≥95th percentile)	33.4	42.2	
**Classification by fasting blood glucose level, mg/dL**			
Normal (<100)	50.6	66.7	.01
Prediabetes (100–25)	24.8	31.1	
Diabetes mellitus (≥126)	25.6	2.2	

a *P* values determined using *t* test.

In a comparison of the baseline and final assessments, 3 physical fitness measures improved for female participants and 1 improved for male participants (Table 4). Girls showed significant improvements in curl-ups (*P* = .01), push-ups (*P* < .001), and the PACER run (*P* = .01). Boys yielded significant improvements in push-ups (*P* = .02).

**Table 4:** Physical Fitness Measures Over Time of Hualapai Youth (N = 71) Participating in a School-Based Intervention to Prevent Diabetes, Arizona, 2004–2006

Measure	Baseline, Mean (SD)	End of Study, Mean (SD)	*P* Value^a^
**No. of curl-ups**			
Male	32.9 (11.8)	36.4 (10.4)	.07
Female	33.3 (9.1)	38.3 (9.1)	.01
**No. of push-ups**			
Male	33.1(15.1)	40.2 (18.5)	.02
Female	32.4 (9.1)	44.9 (11.4)	<.001
**Sit and reach, in**			
Male	9.0 (2.3)	9.6 (2.0)	.13
Female	10.8 (2.4)	11.4 (2.5)	.19
**Pacer run, lap**			
Male	21.9 (16.9)	27.1 (17.4)	.08
Female	18.6 (13.3)	27.4 (15.1)	.01
**Shuttle run, sec**			
Male	13.9 (1.5)	12.8 (1.6)	.99
Female	14.6 (2.0)	13.2 (1.3)	.99

Abbreviation: SD, standard deviation.

a *P* values determined using *t* test.

## Discussion

This service-research intervention depended on a CBPR approach. Tribal, school, parental, and minor consent obtained by the community partners were essential to the implementation of the physical activity classes and the assessment sessions, which involved collecting blood samples from youth. Development of a practical research plan that provided a service to the youth, the school, and the community and that yielded sound scientific outcomes required the integration of community and academic skills. Throughout the 2-year evaluation, the research team recruited and gained minor assent and parental consent for 95% to 100% of the youth in the 5 participating classes. Failure to engage more youth in the assessment sessions was linked to high rates of tardiness, absenteeism, and truancy (estimated as 35% to 45% daily) and to teachers requiring students to use their physical activity sessions to make up missed classroom work. Blood samples were not collected from youth who missed the bus and arrived late, missed school entirely on the days of assessment, or were held out of class; these circumstances yielded a small but useable sample size of approximately 70% of the initial participants.

This project continued without incident despite the research-related law suit filed by the neighboring Havasupai Tribe regarding the misuse of tribal members’ blood samples drawn during a DM study in the 1990s (12). The Hualapai and Havasupai Tribes share a reservation boundary, cultural heritage, and traditions and have frequent social, cultural, and political interaction. At the time of this research, the Hualapai Tribal Council was aware of the Havasupai lawsuit against academic researchers and Arizona State University, a state institution governed by the same board of regents as University of Arizona (12). The long-standing relationship and trust between the tribal and university co-principal investigators of this study, regular communication with the Tribal Council, and the integrity and sociocultural capital of the community researchers were critical to the implementation of the Youth Wellness Program (26).

Many community-driven interventions fail to collect credible quantitative measures and thus struggle to demonstrate the measureable impact of their efforts (7). Given the diversity of DM risk factors identified in the literature, the tribe–university research team selected a range of measures to detect changes in body weight, muscular and cardiovascular fitness, and physiology. The measures selected (BMI, fasting blood glucose level, and 5 items from the Fitnessgram) required personnel training, specialized equipment (ie, blood glucose meter and stadiometer) as well as nonmedical supplies (ie, stretch-and-reach box, timed sound recording, and a stopwatch for the Fitnessgram measures). These items and skills remained within the Tribal Health Department at the end of the study. This project enhanced local capacity for intervention and assessment by demonstrating parents’ receptivity to in-school health assessments that involved a finger stick to collect a blood sample, the ease with which lay personnel could efficiently collect health and fitness measures, and the school administration’s willingness to allow trained Tribal Health Department staff to lead and assess physical activity classes in school. Both the intervention and limited assessment activities (fitness measures only) continue to be implemented by the Hualapai Tribal Diabetes Prevention Program, a program within the Tribal Health Department. In the absence of continued research funding, the cost of the fasting blood glucose measure could not be supported.

The quantitative outcomes of this project support findings in national reviews of health promotion activities in Native populations that increased levels of physical activity can provide a protective effect against DM by reducing the prevalence of selected risk factors (7,27). In this school-based intervention, regular physical activity classes were associated with improved body-core and upper-body fitness, assessed by curl-ups and push-ups, and reduced fasting blood glucose levels. Imperatore et al (28) reported that physical activity stimulates the oxidative enzymes of skeletal muscles and recommended increased physical activity as a means to enhance insulin sensitivity and improve glucose uptake. The association between weight loss and reduced insulin resistance and lower blood glucose levels has been established (29), although we did not find a decrease in BMI among youth in this study. The absence of change in BMI but improved fitness and fasting blood glucose supports Rhee and Woo’s (30) finding that a critical DM risk factor, fasting blood glucose, can be altered without weight loss.

CBPR is an ongoing process that begins with identifying the problem to be addressed and continues through proposing an intervention and research plan, implementing the proposed plan, collecting, analyzing and interpreting data, and disseminating local and national results. The collaborative process is well suited to the development phase of the research project; community and academic team members are enthusiastic about contributing their diverse skills to the design of a creative solution to an ongoing issue. However, once funded, partners may gravitate to specific tasks. Partners should be encouraged to lead the processes in which they have the greatest expertise, but as part of a CBPR team, all partners are obligated to mentor and teach their peers. The goals are to build local and academic capacity and to make a sustainable change to the systems that support improved health.

This study highlights, and in some cases reinforces, valuable elements of the evolving practice of CBPR. The Youth Wellness Program demonstrates that quantitative assessment and research methods are not only valuable but also can be sustained by community programs. The quantitative outcomes of this study were incongruous, and community and academic partners should use a range of indicators sensitive enough to detect change along a broad continuum.
